# Mixed adenoneuroendocrine carcinomas of stomach and ampulla of vater after curative-intent resection: a single center cases series

**DOI:** 10.1186/s12876-021-01909-z

**Published:** 2021-08-25

**Authors:** Sishu Yang, Jiong Lu, Yulong Cai, Bei Li, Xianze Xiong

**Affiliations:** Department of Biliary Surgery, West China Hospital of Sichuan University, No. 37, Guoxue Alley, Chengdu, 610041 Sichuan Province China

**Keywords:** Mixed adenoneuroendocrine carcinomas, Prognosis, Digestive system neoplasms

## Abstract

**Background:**

Mixed adenoneuroendocrine carcinoma is a rare clinical manifestation, especially in the gastric and ampullary. The purpose of this study was to investigate the clinicopathological features and prognosis of mixed adenoneuroendocrine carcinoma in the gastric and ampullary and summarize related treatment suggestions.

**Methods:**

In all, 32 cases of mixed adenoneuroendocrine carcinoma in the gastric and ampullary that were diagnosed from resected specimens were analyzed from 2009 to 2015. The corresponding demographic, clinicopathological and survival data were retrospectively reviewed.

**Results:**

The 1-year, 3-year and 5-year survival rates were 78.1%, 28.1 and 9.4%, respectively, and the median overall survival was 28.0 months. In all, 75.0% (24/32) had lymph node metastasis at the time of initial diagnosis. A multivariate analysis revealed that TNM stage (HR 6.444 95%CI 1.477–28.121 *P* = 0.013), lymph nodes metastasis (HR10.617 95%CI 1.409–79.997 *P* = 0.022), vascular invasion (HR 5.855 95%CI 1.719–19.940 *P* = 0.005), grade of the adenocarcinoma component (HR 3.876 95%CI 1.451–10.357 *P* = 0.007) and CD56 positivity (HR 0.265 95%CI 0.100–0.705 *P* = 0.008) were independent predictors of overall survival.

**Conclusions:**

Mixed adenoneuroendocrine carcinoma is an aggressive clinical entity with a poor prognosis. Taking both the neuroendocrine component and the adenocarcinoma component into consideration of optimal treatment is strongly recommended.

## Background

According to the recent WHO classification from 2010, mixed adenoneuroendocrine carcinomas (MANEC) are composed of both malignant neuroendocrine and exocrine components, and each of them must exceed 30% of the entire tumor cell population [1]. Synaptophysin (Syn), chromogranin (CgA) and CD56 are commonly used neuroendocrine makers, two of which must be positive for a diagnosis of MANEC [2, 3]. MANEC is clinically rare, and its morphology is recognizable as both gland-forming epithelial and neuroendocrine neoplasms [4]. MANECs have been reported in the colon, rectum, esophagus, stomach, and pancreas, among other sites. Although most of them are case reports [5–8], a few studies of MANEC cases in the gastric and ampullary on a much larger scale have been published, the clinicopathological features and prognosis of patients with MANEC in the gastric and ampullary are still unclear, however, there are several researches reported that biological and histological characteristics of MANEC in the stomach and ampulla of vater were similar at some aspect [9–11], most of patients were diagnosed in advanced stages with lymph nodes metastasis, both MANEC in stomach and ampulla of vater are aggressive and lack of effective treatments [12, 13], what’more, p53 nuclear accumulation was detected in most patients of MANEC in stomach and ampulla of vater, and there is a special kind of MANEC with a component “composite glandular and endocrine tumor with pancreatic acinar differentiation” founded in only stomach and ampulla of Vater, which may indicate that there colud be a modicum of similar mechanisms of cancerogenesis in those two sites [4, 10, 14]. In our study, the data of 32 MANEC patients who were treated at West China hospital between 2009 and 2015 were retrospectively analyzed. In this study, we analyzed the clinicopathological features and prognosis of patients with MANEC in the gastric and ampullary and summarized related treatment suggestions according to other recently published literatures.

## Methods

### Study subjects

**Fig. 1:**
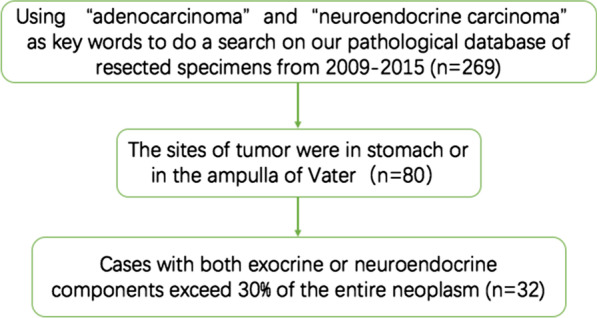
The flowchart of patient selection

**Fig. 2:**
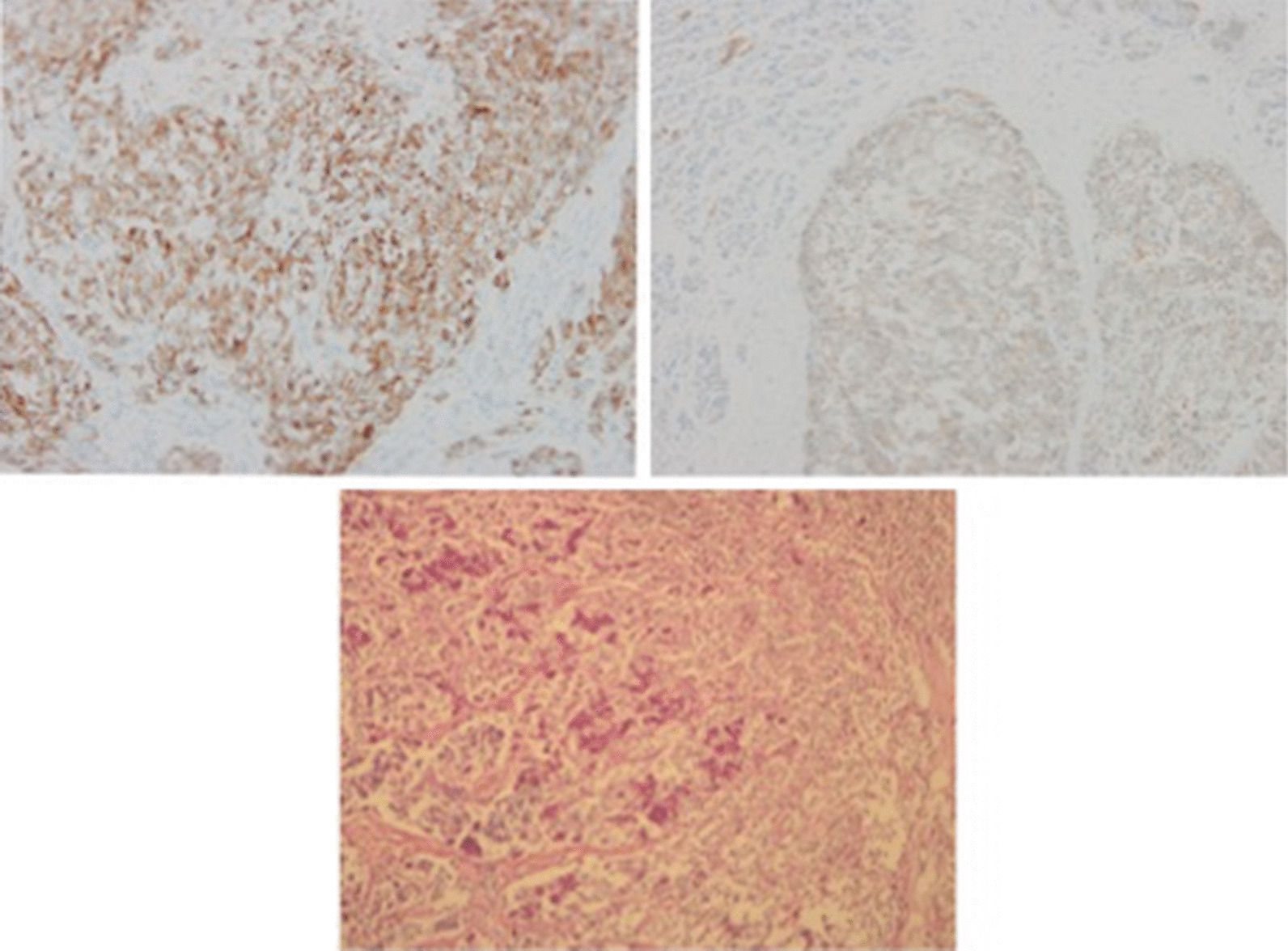
Immunohistochemistry for chromogranin A, original magnification, × 200, B. Immunohistochemistry for Syn, original magnification, × 200, C. Hematoxylin and eosin (H&E) staining, original magnification, × 100

This is a retrospective study. First of all, we choosed “adenocarcinoma” and “neuroendocrine carcinoma” as key words to do a search on our pathological database of resected specimens from 2009 to 2015, then we went through the qualified pathological reports, only the sites of tumor were in stomach or in the ampulla of Vater did the patients are involved in the next step. Finally, tumor that were not composed of adenocarcinoma or a poorly differentiated neuroendocrine carcinoma (G3) were excluded. If either the exocrine or neuroendocrine components did not exceed 30% of the entire neoplasm, cases were also excluded (as showed in Fig. 1). The pathological diagnosis was supported by multiple histologic examinations including hematoxylin and eosin staining and immunohistochemical staining (Fig. 2).

### Clinicopathological data

All the data obtained from the patients was retrospectively reviewed and included age, gender, symptoms, blood type, tumor location, levels of serum alpha fetoprotein (AFP), carbohydrate antigen 19-9 (CA19-9) and carcino embryonic antigen (CEA), ultrasonography, computed tomography, magnetic resonance image and treatments. All the resected specimens were reviewed pathologically and were assessed for tumor size, depth of invasion, pathological classification, histological differentiation, lymph node status, presence of lymphatic and perinerual invasion, cell types of neuroendocrine carcinoma, vascular invasion, Ki-67 index and immunohistochemical staining. The margins were examined for the presence of residual tumor, which was described by the residual tumor classification, as follows: R0, no residual tumor and resection margin was > 0 mm; R1, microscopic residual tumor; R2, macroscopic residual tumor. Tumors were staged according to the American Joint Committee on Cancer (AJCC) 7th TNM staging system [15].

### Follow-up

All the patients received clinical and radiographic follow-up every 2–3 months the first year after the surgery and then 3–6 months annually thereafter. The patients who were at high risk for recurrence received chemotherapy. Follow-up ended December 31, 2017.

### Statistical analysis

Overall survival (OS) was calculated from the time of surgical resection to the time of death. Disease-free survival (DFS) was estimated from the time of surgical resection until the time of relapse confirmed by biopsy or radiologic imaging. Survival curves were generated by the Kaplan–Meier method, and the log-rank test was performed to evaluate the survival difference. All predictors that were statistically correlated with survival in the univariable analysis were included in a multivariable analysis using the Cox proportional regression model. When the *P* value was less than 0.05, the difference was considered statistically significant. SPSS 24.0 statistics software was used for data analysis.

## Results

### Clinical and pathological characteristics

The detailed characteristics are summarized in Table 1. This study included 32 cases of MANEC, including 25 cases in the stomach and 7 cases in the ampulla of Vater. The median age at diagnosis was 59.5 years (range 48–74 years). In all, 78.1% patients were diagnosed with stage III/IV disease, and 75.0% had lymph node metastasis at the time of initial diagnosis. All the patients underwent radical resection with or without neoadjuvant chemotherapy. All of them were R0 resections. Eleven patients (34.4%) received chemotherapy after surgery, 6 patients with MANEC in stomach received etoposide + cisplatin (EP) (54.5%, 6/11) and 5 patients with MANEC in ampulla of Vater received gemcitabine + cisplatin (45.5%, 5/11). The pathological grade of the neuroendocrine component in all cases was G3. The pathological pattern of the adenocarcinoma component was well differentiated (9.3%), moderately differentiated (37.5%), poorly differentiated (31.3%) and signet ring cell/mucin (21.9%). Immunohistochemical staining was positive for chromogranin (81.3%), synaptophysin (96.9%) and CD56 (56.3%), and the positivity rates for pan-cytokeratin (PCK), epithelial membrane antigen (EMA), and CK7 were 56.3%, 34.4 and 25.0%, respectively.

**Table 1:** The clinical and pathological characteristics of MANEC in the gastric and ampullary

Variables	MANEC (n = 32)
Age, years, median (range)	59.5 (48–74)
Sex, male/female, n	26/6
AFP, ng/ml, median (range)	3.23 (1.04–64.65)
AFP > 20 ng/ml, n (%)	2 (6.2%)
CA19-9, U/ml, median (range)	12.82 (0.91–866.8)
CA19-9 > 40 U/ml, n (%)	5 (15.6%)
CEA, ng/ml, median (range)	3.12 (0.93–86.67)
CEA > 8 ng/ml, n (%)	5 (15.6%)
Tumor location, n	
Ampulla of Vater	7
Stomach	25
Tumor size < 3 cm/>3 cm	14/18
T, n (%)	
T1	1 (3.1%)
T2	6 (18.8%)
T3	16 (50.0%)
T4	9 (28.1%)
Lymph node metastasis, n (%)	24(75.0%)
TNM stage*, n (%)	
I	2(15.6%)
II	5 (15.6%)
III	24 (65.7%)
IV	1 (3.1%)
Ki67 median (range)	50.0% (10.0–90.0%)
Cell type of NEC, n (%)	
Large cell	11 (34.4%)
Mixed cell	14 (43.8%)
Small cell	7 (21.8%)
Lymphatic invasion yes/no	27/5
Angioinvasion yes/no	18/14
Perineural invasion yes/no	20/12
Differentiation of adenocarcinoma, n (%)	
Well differentiation	3 (9.3%)
Moderate differentiation	12 (37.5%)
Poor differentiation	10 (31.3%)
Signet ring cell/Mucin	7 (21.9%)
Syn (+), n (%)	31 (96.9%)
CgA (+), n (%)	26 (81.3%)
CD56 (+), n (%)	18(56.3%)
EMA(+), n (%)	11 (34.4%)
PCK(+), n (%)	18 (56.3%)
CK7(+), n (%)	8 (25.0%)
Chemotherapy, n (%)	11 (34.4%)

*MANEC* Mixed adenoneuroendocrine carcinomas

*AJCC TNM staging system of tumors (7th edition, 2010)

### Postoperative recurrence

The deadline for follow-up was December 2017. All 32 patients were followed-up with a median follow-up time of 38.0 months (range 6.0–68.0 months). During the follow-up, 16 (50.0%) patients experienced recurrence. Moreover, 43.8% (7/16) of patients had multiple recurrences, most of which occurred in the retroperitoneal lymph nodes (75.0%, 12/16). The median DFS was 22.0 months. In the univariate analysis, tumor size ≤ 3 cm (*P* = 0.036); lymphatic invasion (*P* = 0.027); grade of the adenocarcinoma component (*P* = 0.000); CD56 (+) (*P* = 0.017); lymph nodes metastasis (*P* = 0.013) and vascular invasion (*P* = 0.006) were defined as predictors of DFS (Table 2). The factor that was statistically significant in the univariate analysis was included in the Cox model. In the multivariate analysis, lymph nodes metastasis (HR 8.380 95%CI 1.096–64.094 *P* = 0.041), vascular invasion (HR 9.923 95%CI 1.298–75.864 *P* = 0.027), grade of the adenocarcinoma component (HR 6.331 95%CI 2.046–19.587 *P* = 0.001), and CD56 positivity (HR 0.318 95%CI 0.112–0.906 *P* = 0.032) were independent prognostic predictor of DFS (Table 2).

**Table 2:** Univariate and multivariate analyses of prognostic factors that influence recurrence of MANEC in the gastric and ampullary

Variables	Univariate analysis			Multivariate analysis	
	No. of patients (%)	MDFS (months)	*P* value	Hazard ratio (95%CI)	*P* value
Age (years)					
< 60	16	20.0	0.402		
≥ 60	16	–			
Sex					
Male	26	–	0.272		
Female	6	15.0			
Tumor location					
Stomach	25	–	0.324		
Ampullary	7	20.0			
Tumor size					
≤ 3 cm	14	–	0.036	1.942 [0.606, 6.223]	0.264
> 3 cm	18	12.0			
T					
T1/T2	7	–	0.030	3.611 [0.335, 38.888]	0.290
T3/T4	25	22.0			
Lymph node metastasis					
No	8	–	0.013	8.380 [1.096, 64.094]	0.041
Yes	24	20.0			
TNM stage					
I/II	10	–	0.015	0.797 [0.082, 7.792]	0.846
III/IV	22	20.0			
Ki67 (%)					
≤ 20	3	22.0	0.638		
> 20	29	26.0			
Cell type of NEC					
Large cell	11	–	0.612		
Non-large cell	21	25.0			
Lymphatic invasion					
Presence	27	22.0	0.027	–	0.975
Absence	5	–			
Vascular invasion					
Presence	23	20.0	0.006	9.923 [1.298, 75.864]	0.027
Absence	9	–			
Perineural invasion					
Presence	22	22.0	0.364		
Absence	10	–			
Grade of adenocarcinoma					
Well/moderate differentiation	20	–	0.000	6.331 [2.046, 19.587]	0.001
Poor/signet ring cell/mucin	12	9.0			
Syn					
(+)	31	25.0	0.397		
(−)	1	–			
CgA					
(+)	26	26.0	0.668		
(−)	6	25.0			
CD56					
(+)	18	–	0.017	0.318 [0.112, 0.906]	0.032
(−)	14	9.0			
PCK					
(+)	18	26.0	0.913		
(−)	14	22.0			
EMA					
(+)	11	25.0	0.713		
(−)	21	26.0			
CK7					
(+)	8	20.0	0.515		
(−)	24	26.0			
Chemotherapy					
Yes	11	26.0	0.71		
No	21	25.0			

*MANEC* Mixed adenoneuroendocrine carcinomas

*AJCC TNM staging system of tumors (7th edition, 2010)

### Overall survival

The median overall survival was 28.0 months. The 1-year, 3-year and 5-year survival rates were 78.1%, 28.1 and 9.4%, respectively. In the univariate analysis, tumor size ≤ 3 cm (*P* = 0.001), lymphatic invasion (*P* = 0.018), vascular invasion (*P* = 0.006), lymph node metastasis (*P* = 0.004), TNM stage (*P* = 0.004), grade of the adenocarcinoma component (*P* = 0.003) and CD56 positivity (*P* = 0.006) were associated with the outcome (Table 3). In the multivariate analysis, TNM stage(HR 6.444 95%CI 1.477–28.121 *P* = 0.013), lymph node metastasis (HR 10.617 95%CI 1.409–79.997 *P* = 0.022), vascular invasion (HR 5.855 95%CI 1.719–19.940 *P* = 0.005), grade of the adenocarcinoma component (HR 3.876 95%CI 1.451–10.357 *P* = 0.007) and CD56 positivity (HR 0.265 95%CI 0.100–0.705 *P* = 0.008) were independent predictors of overall survival (Table 3).

**Table 3:** Univariate and multivariate analyses of prognostic factors that influence overall survival of MANEC in gastric and ampullary

Variables	Univariate analysis			Multivariate analysis	
	No. of patients (%)	MOS (months)	*P* value	Hazard ratio (95%CI)	*P* value
Age (years)					
< 60	16	24.0	0.297		
≥ 60	16	42.0			
Sex					
Male	26	28.0	0.539		
Female	6	24.0			
Tumor location					
Stomach	25	25.0	0.644		
Nonstomach	7	28.0			
Tumor size					
** ≤** 3 cm	14	–	0.001	3.006 [0.992, 9.107]	0.052
> 3 cm	18	15.0			
T					
T1/T2	7	–	0.006	6.089 [0.549, 67.571]	0.141
T3/T4	25	24.0			
Lymph node metastasis					
No	8	–	0.004	10.617 [1.409, 79.997]	0.022
Yes	24	18.0			
TNM stage					
I/II	10	–	0.004	6.444 [1.477, 28.121]	0.013
III/IV	22	18.0			
Grade of adenocarcinoma					
Well/moderate differentiation	20	42.0	0.003	3.876 [1.451, 10.357]	0.007
Poor/signet ring cell/mucin	12	12.0			
Ki 67(%)					
≤ 20	3	24.0	0.919		
> 20	29	28.0			
Cell type of NEC					
Large cell	11	25.0	0.956		
Non-large cell	21	28.0			
Lymphatic invasion					
Presence	27	24.0	0.018	–	0.972
Absence	5	–			
Vascular invasion					
Presence	18	24.0	0.006	5.855 [1.719, 19.940]	0.005
Absence	14	–			
Perineural invasion					
Presence	20	24.0	0.450		
Absence	12	30.0			
Syn					
(+)	31	25.0	0.669		
(−)	1	28.0			
CgA					
(+)	26	25.0	0.496		
(−)	6	28.0			
CD56					
(+)	14	30.0	0.006	0.265 [0.100, 0.705]	0.008
(−)	18	12.0			
PCK					
(+)	18	25.0	0.736		
(−)	14	28.0			
EMA					
(+)	11	25.0	0.657		
(−)	21	28.0			
CK7					
(+)	8	24.0	0.915		
(−)	24	25.0			
Chemotherapy					
Yes	11	24.0	0.758		
No	21	28.0			

*MANEC* Mixed adenoneuroendocrine carcinomas

*AJCC TNM staging system of tumors (7th edition, 2010)

## Discussion

MANEC is clinically rare since only when the exocrine and neuroendocrine components each exceed 30% can the tumor be considered as MANEC. Gastric and ampullary MANEC are highly exceptional entities [13, 16, 17]. To the best of our knowledge, this research is the largest case series of MANEC in the gastric and ampullary.

Three commonly used neurendocrine makers, Syn (96.9%), CgA (81.3%) and CD56 (56.3%), were all observed in our study. This result is similar to that of the study by Brathwaite et al. in that Syn and CgA were expressed in 97 and 82% of cases, respectively 18]. A previous report indicated that several markers, including CK7 and CK20, detected by immunohistochemical staining did not reveal any correlation with prognosis in MANECs [19]. However, regarding hematopoietic stem cell markers, immunoreactivity for CD117 has already been considered an indicator of malignancy in pancreatic neuroendocrine carcinomas [20–23]. In our study, we found that although CD56 was not as sensitive as CgA (81.3%) and Syn (96.9%), CD56 demonstrated a strong association with overall survival. CD56 is an important marker of neuroendocrine tumors and is also a glycoprotein of the immunoglobulin (Ig) superfamily expressed on natural kill (NK) cells, NK-T cells, and in vitro-expanded cytokineinduced cells [24]. It had been shown that CD56^+^ cells exhibited cytotoxic effect against tumor targets [25]. In our study, the OS of the CDK56-positive group was much longer than that of the CD56-negative group (30.0 months vs. 12.0 months) (*P* = 0.006). In the multivariate analysis, CD56 positivity (HR 0.265 95%CI 0.100, 0.705 *P* = 008) was an independent prognostic predictor of OS. This may be related to the cytotoxic effect of CD56^+^ cells. However, CD56 was also expressed in a subset of biliary epithelial cells, especially in intrahepatic small bile ducts, and thus it might not be the best marker of neuroendocrine tumors in the biliary duct [26].

In this study, more than half of the patients presented with perinerual invasion (13/32, 61.9%) or vascular invasion (13/32, 61.9%), which is a little lower than Wantanabe’s and La Rosa’s results [19, 27]. In our study, vascular invasion (HR 5.855 95%CI 1.719–19.940 *P* = 0.005) was one of the independent predict factors on overall survival, which was consistent with La Rosa’s conclusion that vascular invasion had a negative effect on the prognosis [19]. There were 84.4% of patients with the presence of the lymphatic invasion and it was a predict factor of bad outcome in univariate analysis (*P* = 0.018). which was disagreement with the La Rosa’s result that the patients with the presence of lymphoid infiltrate had a better prognosis [19]. But both of two analysis had a limited case samples of MANECs and were lost in multivariate analysis. Hence, further studies are needed on impact of lymphatic invasion.

The optimal treatment of MANEC is still controversial. Most published studies have indicated that the treatment should be based on the most aggressive histologic component [4, 16, 28], but which histologic component is the most aggressive? Some research has focused on this question. Chen et al. reported that the pure neuroendocrine component accounts for a large proportion (6/9) of lymph node metastasis in MANEC patients, and patients who have died had a predominantly neuroendocrine component in the primary tumor or in metastatic lymph nodes[29]. This finding was in agreement with that of the study by Harada et al. [30]. They found that at the surface of the tumor, the adenocarcinoma components were in the highest flight. Neuroendocrine components are involved in most stromal and vascular invasion and LN metastasis. Moreover, neuroendocrine components showed higher proliferative activity than adenocarcinoma components [30, 31], which suggested that the neuroendocrine component was more aggressive and defined the prognosis of MANEC [32]. However, in our study, we excluded all the patients with neuroendocrine tumors (G1/G2) according to the definition of MANEC by WHO [1], and we found that patients with well/moderately differentiated adenocarcinoma had a better prognosis. In the multivariate analysis, the grade of the adenocarcinoma component (HR 3.876 95%CI 1.451–10.357 *P* = 0.007) was an independent predictor of overall survival, which was agreed with the conclusion of Nie’s research [17]. Hence, we suggested that the optimal treatment of MANEC should take both the neuroendocrine component and the adenocarcinoma component into consideration.

Several limitations of our study should be noted. Our data were retrospectively collected from a single medical center, which may carry an inherent risk of bias. The small sample size in this study also limited our ability to conduct further analyses. Despite these limitations, this study reflected the actual clinical features and prognostic factors of MANEC of the gastric and ampullary.

## Conclusions

Overall, MANEC is a highly aggressive clinical entity with a poor prognosis. CD56 negativity may reveal a high risk for recurrence. Tumor size, lymph node metastasis and grade of the adenocarcinoma component were independent predictors of overall survival, hence, the grade of the adenocarcinoma component should be taken into consideration of the treatments as same as the neuroendocrine component.

## Data Availability

The datasets generated and/or analysed during the current study are available from the corresponding author on reasonable request.

## Abbreviations

MANEC: Mixed adenoneuroendocrine carcinoma
Syn: Synaptophysin
CgA: Chromogranin
AFP: Alpha fetoprotein
CA19-9: Carbohydrate antigen
CEA: Carcino-embryonic antigen
DFS: Diease free survival
OS: Overall survival
PCK: Pan-cytokeratin
EMA: Epithelial membrane antigen
NK cell: Natural kill cell
